# Adiponectin Gene Variant rs3774261, Effects on Lipid Profile and Adiponectin Levels after a High Polyunsaturated Fat Hypocaloric Diet with Mediterranean Pattern

**DOI:** 10.3390/nu13061811

**Published:** 2021-05-26

**Authors:** Daniel Antonio de Luis Roman, David Primo, Olatz IZaola, Emilia Gómez, Juan Jose López

**Affiliations:** Center of Investigation of Endocrinology and Nutrition, Department of Endocrinology and Investigation, Medicine School, Hospital Clinico Universitario, University of Valladolid, 47005 Valladolid, Spain; dprimoma@saludcastillayleon.es (D.P.); oizaolaj@saludcastillayleon.es (O.I.); egomezho@saludcastillayleon.es (E.G.); jlopezgo@saludcastillayleon.es (J.J.L.)

**Keywords:** adiponectin, dietary intervention, insulin resistance, obesity, rs3774261

## Abstract

The role of *ADIPOQ* gene variants on metabolic improvements after weight change secondary to different hypocaloric diets remained unclear. We evaluate the effect of rs3774261 of *ADIPOQ gene* polymorphism on biochemical improvements and weight change after high polyunsaturated fat hypocaloric diet with a Mediterranean dietary pattern for 12 weeks. A population of 361 obese subjects was enrolled in an intervention trial with a calorie restriction of 500 calories over the usual intake and 45.7% of carbohydrates, 34.4% of fats, and 19.9% of proteins. The percentages of different fats was; 21.8% of monounsaturated fats, 55.5% of saturated fats, and 22.7% of polyunsaturated fats. Before and after intervention, an anthropometric study, an evaluation of nutritional intake and a biochemical evaluation were realized. All patients lost weight regardless of genotype and diet used. After 12 weeks with a similar improvement in weight loss (AA vs. AG vs. GG); total cholesterol (delta: −28.1 ± 2.1 mg/dL vs. −14.2 ± 4.1 mg/dL vs. −11.0 ± 3.9 mg/dL; *p* = 0.02), LDL cholesterol (delta: −17.1 ± 2.1 mg/dL vs. −6.1 ± 1.9 mg/dL vs. −6.0 ± 2.3 mg/dL; *p* = 0.01), triglyceride levels (delta: −35.0 ± 3.6 mg/dL vs. 10.1 ± 3.2 mg/dL vs. −9.7 ± 3.1 mg/dL; *p* = 0.02), C reactive protein (CRP) (delta: −2.3 ± 0.1 mg/dL vs. −0.2 ± 0.1 mg/dL vs. −0.2 ± 0.1 mg/dL; *p* = 0.02), serum adiponectin (delta: 11.6 ± 2.9 ng/dL vs. 2.1 ± 1.3 ng/dL vs. 3.3 ± 1.1 ng/dL; *p* = 0.02) and adiponectin/leptin ratio (delta: 1.5 ± 0.1 ng/dL vs. 0.3 ± 0.2 ng/dL vs. 0.4 ± 0.3 ng/dL; *p* = 0.03), improved only in AA group. AA genotype of *ADIPOQ variant* (rs3774261) is related with a significant increase in serum levels of adiponectin and ratio adiponectin/leptin and decrease on lipid profile and C-reactive protein (CRP).

## 1. Introduction

The pandemic of obesity has been termed “globesity” to remark the global nature of the problem. Lifestyle modifications with a low-calorie diet produce weight loss and the secondary improvement of many of the components of associated comorbidities, including hyperlipidemia, diabetes mellitus type 2, hypertension, and inflammatory markers [1]. Now, adipose tissue has been considered an important cornerstone endocrine organ secreting several adipokines implied in the regulation of metabolism and energy status. Some adipokines have a proinflammatory role as leptin and resistin and other groups such as adiponectin has an anti inflammatory function [2]. Adiponectin is the most important adipokine secreted by this tissue [3]. Adiponectin has an anti-inflammatory role and its levels are reduced in obese subjects and are enhanced after weight reduction [4]. Low adiponectin levels have been related with a high risk of obesity, diabetes mellitus, and hyperlipidemia [5], with a potential therapeutic effect with agonists of adiponectin [6].

The adiponectin levels are highly heritable, and the *ADIPOQ* gene is the principal locus promoting variations in serum levels [7]. Single nucleotide polymorphisms (SNPs) are genetic variants that can sometimes have functional implications in the *ADIPOQ* gene, which is situated on chromosome 3q27. One of these SNPs, 712 G/A rs3774261 in the *ADIPOQ* has been related to diabetes mellitus in obese subjects [7] and with coronary heart disease [8,9]. Interestingly, this genetic variant has been associated with eating behavior [10], and there are nutritional intervention studies [11,12], too. These interventional designs [11,12] reported significant results on serum lipid profile and inflammatory markers. Our previous study with a Mediterranean diet [11] had a lower percentage of fat in the diet than the current intervention and low sample size; with this new study, we will evaluate more precisely the effect of the quantity and quality of fat in a larger sample of patients. Perhaps the metabolic effects found in these studies and their relationship with this genetic variation are due to both factors; weight loss and the Mediterranean diet pattern used. The beneficial effects of a diet with a Mediterranean style can be due to the presence of different foods and nutrients such as type of dietary unsaturated fatty acids [13]. Unsaturated fatty acids are ligands for the transcription factor PPAR gamma [14], which increases *ADIPOQ* gene expression and improves adiponectin concentration [15]. Perhaps increasing the amount of unsaturated fat in a hypocaloric diet would have greater benefits than a conventional hypocaloric diet and rs3774261 would modulate these changes. In previous studies [11,12], the beneficial effects found with the Mediterranean diet were related to the high consumption of olive oil, and therefore, of monounsaturated fatty acids. Notwithstanding that the polyunsaturated fatty acids may also play a relevant role and interact with this genetic variant of the ADIPOQ gene, this hypothesis has not yet been evaluated.

Given this lack of information, we conducted a study to evaluate the effects of a high polyunsaturated fat hypocaloric diet with a Mediterranean style during 12 weeks on metabolic changes considering the rs3774261 of *ADIPOQ*.

## 2. Subjects and Methods

### 2.1. Subjects and Clinical Investigation

Obese subjects were enrolled by the primary care physicians of our health area to treat obesity. These subjects were evaluated in a single-arm clinical trial with a high fat polyunsaturated hypocaloric diet with a dietary Mediterranean pattern. The local ethics committee (Hospital Clinico Universitario Valladolid committee 7/2017, code: GRS588/A/11) approved the protocol; it was in accordance with the guidelines laid down in the Declaration of Helsinki and all subjects gave written informed consent. Clinical and biochemical variables were recorded at the beginning and after 12 weeks of dietary interventions. All the enrolled obese subjects met the following inclusion criteria; age between 30 and 60 years old and an obesity category as a body mass index (BMI) ≥ 30 kg/m^2^. The exclusion criteria were any of the following data: previous cardiovascular event, chronic renal failure, chronic liver failure, alcoholism, malignant tumor, and within the 24 weeks before the study were taking any medications or nutrient-supplements or have been on a low-calorie diet.

The main objective of our study was serum adiponectin change after 12 weeks versus baseline. The secondary objectives were improvements in lipid profile and glucose metabolism after dietary intervention. Lipid profile (LDL-cholesterol, HDL-cholesterol, triglycerides, and total cholesterol), C-reactive protein (CRP), insulin, and adipokines (resistin leptin and total adiponectin) levels were analyzed. Homeostasis model assessment (HOMA-IR) and adiponectin/leptin ratio were calculated, too. The anthropometric evaluation was realized with body weight, height, waist circumference, fat mass by bioimpedance, and calculated body mass index (BMI). Systolic and diastolic blood pressure was recorded. All clinical and biochemical parameters were determined at the basal time and after 3 months of dietary intervention. The genetic variant rs3774261 of the *ADIPOQ* gene was assessed.

### 2.2. Dietary Intervention

A total of 361 obese patients met the above-mentioned criteria, and they were included to observe a hypocaloric diet for 12 weeks. The diet (high-polyunsaturated fatty acid hypocaloric diet Mediterranean diet) was based on a calorie restriction of 500 calories over the usual intake, 45.7% of carbohydrates, 34.4% of fats, and 19.9% of proteins. The percentages of different fats were: 21.8% of monounsaturated fats, 55.5% of saturated fats, and 22.7% of polyunsaturated fats (13 g per day of w-6 fatty acids, 3 g per day of w-3 fatty acids, and a ratio w6/w3 of 4.3).

Food tables were used with a Mediterranean dietary style, including (legumes, vegetables, and fresh fruit 5 servings per day, poultry, whole grains, fish 3 times per week, using 20 g olive oil per day, 40 g of walnuts daily, and limit unhealthy fats such as margarine, fatty meats, snacks, industrial pastries) [16]. To improve compliance with dietary intervention, the completion of diet recommendations was evaluated every 10 days with a phone call. Records of daily dietary intake for 4 days were parameterized with software (Dietosource^®^, Geneva, Switzerland), and this software is based on national composition food tables [15]. The recommendations for physical activity were aerobic physical activities at least 3 times each week (60 min each). The physical activity allowed by protocol were (cycling, running, walking, and swimming). Each patient with a self-reported questionnaire recorded the physical activity.

### 2.3. Biochemical Parameters

Blood samples were drawn after a minimum of 10 h overnight and these samples were stored at −80 °C until analyzed. Lipid profile (low-density lipoprotein (LDL)-cholesterol, high-density lipoprotein (HDL)-cholesterol, triglycerides, and total cholesterol), C reactive protein (CRP), fasting glucose, and insulin levels were determined on the same day using the clinical chemistry automated analyzer COBAS INTEGRA 400 analyzer (Roche Diagnostic, Montreal, Canada). LDL cholesterol was calculated using the Friedewald formula (LDL cholesterol = total cholesterol-HDL cholesterol-triglycerides/5) [17]. Insulin resistance was calculated using the homeostasis model assessment (HOMA-IR) with the following equation (glucosexinsulin/22.5) [18].

Serum adipokines were measured by enzyme-linked immunosorbent assays (ELISA). Resistin kit had with a normal range of 4–12 ng/mL [19] (Biovendor Laboratory, Inc., Brno, Czech Republic). The leptin kit had a normal range of 10–100 ng/mL (Diagnostic Systems Laboratories, Inc., Webster, TX, USA) [20]. Finally, the adiponectin kit had a normal range of 8.65–21.43 ng/mL (R&D Systems, Inc., Minneapolis, MN, USA) [21]. Adiponectin/leptin ratio was calculated in all samples.

### 2.4. Genotyping ADIPOQ Gene

The genotype of SNP rs3774261 of *ADIPOQ* was determined with a polymerase chain reaction in real-time from peripheral blood leucocytes. Genomic DNA was obtained from a 150 uL buffy coat using a blood genomic kit (Bio-Rad^®^, Hercules, CA, USA) in accordance with the manufacturer´s instructions. Probes and oligonucleotide primers and were designed with the Beacon Designer 5.0 (Premier Biosoft International^®^, LA, CA, USA). The polymorphic region of adiponectin was amplified using the polymerase chain reaction (PCR) with 50 ng of this genomic DNA, with allele-specific sense primers (primer forward: 5’-ACGTTGGATGCTCCTCCTTGAAGCCTTCAT-3’ and reverse 5’-ACGTTGGATGCAAGTATTCAAAGTATGGAGC-3’ in a 2 µL final volume (Termocicler Life Technologies, LA, CA, USA). Cycling parameters were as follows: after DNA denaturation at 95 °C for 1 min and annealing at 65 °C for 30 s. The PCR was run in a 25 µL final volume containing 10.5 µL of IQTM Supermix (Bio-Rad^®^, Hercules, CA, USA) with hot start Taq DNA polymerase. Duplicates in the arrays were the methodology to internal controls and the accuracy. Hardy Weinberg equilibrium was determined with a statistical test (Chi-square) to compare our expected and observed counts. The variant was in Hardy Weinberg equilibrium (*p* = 0.31).

### 2.5. Anthropometric Parameters and Blood Pressure

Bodyweight, height, and waist circumference (WC) were determined in the morning before breakfast at baseline and after 3 months. Body mass index was determined by the equation (weight in kg divided by the height in meters squared). Bodyweight was determined with a scale (Omron, LA, CA, USA), and the obese subjects were minimally unclothed to the nearest 0.1 kg and not wearing shoes (Omron, LA, CA, USA). Fat mass was estimated by bioimpedance (Akern, EFG, Pisa, Italy) with an accuracy of 50 g [22]. WC was measured with a measuring tape in the narrowest diameter between the xiphoid process and iliac crest. Systolic and diastolic blood pressures were measured three times and averaged after a 5 min rest with a random zero mercury sphygmomanometer, (Omron, LA, CA, USA).

### 2.6. Statistical Analysis

We used the software SPSS for Windows, version 23.0 software package (SPSS Inc. Chicago, IL, USA) to analyze the data. The sample size was determined to assess changes over 5 ng/mL of adiponectin levels with 90% power and 5% significance (*n* = 300). Results were expressed as average ± standard deviation. Each variable was evaluated for normality with the Kolmogorov–Smirnov test. The parametric test was investigated with the ANOVA test and Bonferroni post hoc test. Non-parametric parameters were evaluated with the Mann-Whitney U-test. Categorical variables were revised with the chi-square test, with Yates correction as necessary, and Fisher’s test. The gene–diet interaction was assessed with a univariate ANCOVA adjusted by gender, and baseline weight. Correction for multiple hypotheses testing for single SNP analyses was performed. A Chi-square test was used to determine the Hardy–Weinberg equilibrium. A *p*-value < 0.05 was considered significant.

### 2.7. Ethical Approval

All methodology of our study were in accordance with the ethical standards of the institutional and/or national research committee (Hospital Clinico Universitario Valladolid committee 7/2017, code: GRS588/A/11) and with the 1964 Helsinki declaration and its later amendments or comparable ethical standards. Informed consent was signed from all individual participants included in the study.

## 3. Results

### 3.1. Characteristics of Participants and Dietary Intakes

We evaluated the role of SNP rs3774261 on the modification of adiposity markers and biochemical variables in 361 obese outpatients. The average age of the sample was 47.1 ± 3.1 years (range: 29–63) and the average body mass index (BMI) was 37.3 ± 4.9 kg/m^2^ (range: 33.5–49.9). Sex distribution was 259 females (71.7%) and 102 males (28.3%). The genotype distribution of this sample was as follows: 117 patients (32.4%) AA, 164 patients AG (45.4%), and 80 patients GG (22.2%). Allelic frequency was 0.62 A and 0.38 G. Sex distribution and the average age was similar in all genotype groups (Table 1). 

Following the sessions of the dietitian, the dietary recommendations were reached at 12 weeks in all genotype groups with a significant decrease of total caloric amount, carbohydrates, fats and proteins (Table 1). A significant increase was observed in the percentage of monounsaturated and polyunsaturated fats (Table 1).

At basal time, the physical activity was similar in the three groups (Table 1). In addition, after the intervention, the physical activity improved, but this improvement did not show differences in total quantity deltas (AA vs. AG vs. GG) (28.2 ± 1.2 min/week vs. 29.8 ± 2.1 min/week vs. 29.1 ± 1.1 min/week; *p* = 0.52).

### 3.2. Anthropometric Results

For rs3774261, there were no statistical differences in anthropometric parameters and systolic/diastolic blood pressure in basal and post-intervention values (AA vs. AG vs. GG) (Table 1). After a high polyunsaturated fat hypocaloric diet with a Mediterranean style, we observed a significant improvement of body mass index, weight, waist circumference, fat mass, and systolic blood pressure. These statistically significant changes were similar in all genotype groups. Diastolic blood pressure remained unchanged.

#### 3.2.1. Biochemical Parameters

In the second analysis of our design, we evaluated the actions of this dietary intervention on glucose metabolism, C Reactive protein and lipid profile (Table 2). After a significant body weight loss (AA vs. AG vs. GG); insulin levels (delta: −3.7 ± 0.2 UI/L vs. −3.8 ± 0.3 UI/L vs. −3.6 ± 0.2 UI/L; *p* = 0.33) and HOMA-IR (delta: −1.3 ± 0.2 units vs. −1.2 ± 0.3 units vs. −1.1 ± 0.4 units; *p* = 0.36) improved in all genotypes without intergroup differences. Finally, after 12 weeks (AA vs. AG vs. GG); total cholesterol (delta: −28.1 ± 2.1 mg/dL vs. −14.2 ± 4.1 mg/dL vs. −11.0 ± 3.9 mg/dL; *p* = 0.01), LDL cholesterol (delta: −17.1 ± 2.1 mg/dL vs. −6.1 ± 1.9 mg/dL vs. −6.0 ± 2.3 mg/dL; *p* = 0.01), triglyceride levels (delta: −35.0 ± 3.6 mg/dL vs. 10.1 ± 3.2 mg/dL vs. −9.7 ± 3.1 mg/dL; *p* = 0.03) and C reactive protein (CRP) (delta: −2.3 ± 0.1 mg/dL vs. −0.2 ± 0.1 mg/dL vs. −0.2 ± 0.1 mg/dL; *p* = 0.01) improved only in the AA group.

#### 3.2.2. Adipokine Levels

Table 3 reports changes on serum adipokines and ratio adiponectin/leptin. After dietary intervention, in the AA genotype group (AA vs. AG vs. GG), serum adiponectin (delta: 11.6 ± 2.9 ng/dL vs. 2.1 ± 1.3 ng/dL vs. 3.3 ± 1.1 ng/dL; *p* = 0.01) improved. Adiponectin/leptin ratio improved in the AA genotype group (delta: 1.5 ± 0.1 ng/dL vs. 0.3 ± 0.2 ng/dL vs. 0.4 ± 0.3 ng/dL; *p* = 0.02). In all genotype groups, leptin decreased in a significant way. Serum resistin levels did not change after the dietary intervention.

## 4. Discussion

We revealed, in this study on a high-polyunsaturated fat hypocaloric diet with a Mediterranean dietary pattern, a decline in LDL-cholesterol, triglycerides, and C reactive protein (CRP) and a rise in serum adiponectin levels and adiponectin/leptin ratio that were statistically significant in obese subjects with the AA genotype of rs3774261. In addition, all subjects in all genotype groups showed a significant decline of adiposity parameters and systolic blood pressure after dietary intervention.

In the literature, some investigations have shown the relationship between this genetic variant (rs3774261) on *ADIPOQ* gene and obesity, metabolic syndrome, diabetes mellitus, and serum adiponectin levels [23,24], with an increased risk to present type 2 diabetes, obesity, and hypoadiponectinemia in a non-Caucasian population [24,25]. In addition to this association with high cardiovascular risk pathology, the G allele of rs3774261 *ADIPOQ* has also been related to coronary heart disease [8]. The exact pathways by which genetic variants in the *ADIPOQ* gene produce coronary heart disease is not well known. Moreover, adiponectin, the adipokine encoded by this gene, has been reported to have anti-inflammatory properties that have important effects in fighting against atherosclerosis [26].

There is little information in the literature about the effect of nutritional treatment and this genetic variant. A recent study with a normal hypocaloric Mediterranean diet reported [11] better changes in lipid profile, CRP, and adiponectin levels in subjects with AA genotype compared in a dominant model with (AG + GG). This previous study [11] had a small sample size (*n* = 135) and a separate analysis of the three genotypes could not be performed. In another design with 284 Caucasian obese subjects [12] reported that after a high-fat hypocaloric diet, the response of lipid levels, CRP, and adiponectin was better on AA genotype than GA or GG genotypes, as our present results. The amount of lipid profile improvement was similar in both studies [11,12] and our present study; however, the rise in adiponectin levels and the decline in CRP was two times greater in the study with a high-fat diet [12] than in the other one [11]. In addition, these changes in lipids and CRP are of a similar magnitude to those found in the current study with a high-polyunsaturated fat hypocaloric diet. The caloric restriction was similar in these three studies, about 500 calories to the previous intake. Although both strategies were carried out with a Mediterranean diet pattern, it is important to remark that in the last study [12] and the present study, the percentage of fat in the diet was higher than the old one; 38% [12] and 34% (current) vs. 25% [11]. In our investigation, patients reached a daily intake of almost 30 g of monounsaturated fat and 16 of polyunsaturated fat, compared to 14 g and 3.6 g [11], and 25 g and 13 g [12], respectively.

This relationship of the quality of dietary fats with the biochemical changes due to weight loss and its interaction with this genetic variate of the ADIPOQ gene could be explained with some findings in the literature with other SNPs in this gene. For example, Alsahel et al. [27] showed that the genetic variant (-1006G/A) increased serum adiponectin after a high-monounsaturated diet, whereas in A-allele carriers it is decreased. This differential response was not detected with a low-fat hypocaloric diet. Moreover, the molecular mechanism could be related to the potential action of dietary unsaturated fat as ligands of PPAR gamma. The role of polyunsaturated fatty acids is being evaluated in the literature, and more intervention studies are recommended with known doses of fatty acids to evaluate their effect on adipokines [28]. In addition, this metabolic way could explain our findings on inflammatory status with CRP levels. The NF-kB pathway in the endothelium, which would produce CRP; was inhibited by adiponectin [29], for example, adiponectin contributes more powerfully to CRP elevation than for example smoking habit, age, and other metabolic parameters [29].

It seems clear in the literature that this genetic variance is related to a pro-inflammatory status, not only related to ischemic heart disease [8] but also with ischemic stroke patients [30]. In our interventional design, the response of insulin resistance was similar in all genotypes. Moreover, some studies have reported that patients carrying the A allele have better insulin sensitivity demonstrated by the euglycemic clamp [31]. The response of triglycerides found in our trial may also be important since the association between rs3774261 and coronary heart disease [8] was influenced by interactions with serum triglycerides [8]. We must not forget the differentiated response of adiponectin levels as a function of genotype found in our study to explain our metabolic findings. A novelty finding in our study was the association of this genetic variant with the adiponectin/leptin ratio and its secondary modifications to the diet. The adiponectin/leptin ratio is a biomarker of adipose tissue dysfunction and inflammation [32], which related leptin as an adipokine related to the degree of adiposity [33]. Moreover, an adiponectin/leptin quotient higher than the unit is considered as normal whereas a ratio below or near to 0.5 units may show an increase in the metabolic risk [34], as reported by the three genotypes in our study before weight loss and near to 0.5 in GA and GG genotypes after dietary intervention.

In addition to the relationship between inflammatory pathways and adiponectin response to explain our findings, unknown genetic mechanisms may also be implicated. The rs3774261 variant is in an intron located, a non-coding region. Moreover, the genetic variant in non-coding regions may alter gene splicing, transcription product binding, mRNA deterioration, and gene expression. Thus, any of these above-mentioned mechanisms can be possible pathways to explain our findings. For example, with different levels of adiponectin circulating in a case-control study, a relationship of the G allele of this genetic variant with the prevalence of metabolic syndrome was found [35]. All these relationships are complicated by the existence of a relevant role of adiponectin at the level of the central nervous system in the pathways of hunger and satiety. Recently it has been observed that the rs3774261 variant is related to disinhibition in eating behaviors [10] influencing daily total food uptake.

There are some limitations of this study. First, we only evaluated one SNP of *ADIPOQ*, so other variants could be related to metabolic parameters. Moreover, some synthetic associations of specific unusual variants may be in partial linkage disequilibrium with usual variants as rs3774261. Second, the lack of a control group without diet might be a bias. It might provide more valuable information on the relationship of *ADIPOQ variants* (rs3774261) with metabolic parameters if the comparative study was conducted on an unrelated control population. Third, the self-reported dietary intake of energy and macronutrients is not reliable and it might include bias of under-or over-reporting. Finally, we studied a Caucasian population, and extrapolation to other populations is not possible.

## 5. Conclusions

In conclusion, the AA genotype of the *ADIPOQ variant* (rs3774261) is related to a significant increase in serum levels of adiponectin and ratio adiponectin/leptin and decrease in the lipid profile and C-reactive protein (CRP) after a hypocaloric high polyunsaturated fat Mediterranean diet.

## Figures and Tables

**Table 1:** Changes in anthropometric parameters, dietary intakes, and physical activity rs3774261 (mean ± S.D).

	AA (*n* = 117)		AG (*n* = 164)		AG (*n* = 80)		
	0 time	At 12 weeks	0 time	At 12 weeks	0 time	At 12 weeks	*p* Time AA Time AG Time GG Basal Genotype 12 weeks Genotype
Age	46.3 ± 4.1	-	47.3 ± 4.0	-	46.9 ± 2.3	-	*p* = 0.46 Basal genotype
Gender/male/female%)	76.1/23.9%	-	73.2/26.8%	-	71.7/28.3%	-	*p* = 0.43 Basal genotype
BMI	37.9 ± 4.1	35.9 ± 3.8 *	37.8 ± 4.0	36.1 ± 3.4 *	38.0 ± 2.0	36.3 ± 2.9 *	*p* = 0.01 *p* = 0.02 *p* = 0.01 *p* = 0.46 *p* = 0.43
Weight (kg)	96.4 ± 2.1	92.4 ± 3.0 *	97.5 ± 2.1	93.1 ± 1.4 *	97.3 ± 2.1 *	93.4 ± 2.2 *	*p* = 0.01 *p* = 0.01 *p* = 0.02 *p* = 0.45 *p* = 0.11
Fat mass (kg)	39.8 ± 2.1	36.4 ± 2.0 *	39.7 ± 2.0	36.0 ± 2.1 *	39.8 ± 2.0 *	36.2 ± 1.1 *	*p* = 0.02 *p* = 0.03 *p* = 0.03 *p* = 0.39 *p* = 0.31
WC (cm)	113.1 ± 7.0	109.2 ± 5.2 *	113.4 ± 6.1	109.1 ± 4.1 *	116.2 ± 4.0 *	111.7 ± 3.9 *	*p* = 0.03 *p* = 0.04 *p* = 0.02 *p* = 0.35 *p* = 0.59
SB (mmHg)	127.1 ± 5.1	123.2 ± 6.0 *	127.8 ± 5.0	122.3 ± 4.1 *	126.2 ± 4.0 *	123.1 ± 3.1 *	*p* = 0.02 *p* = 0.03 *p* = 0.02 *p* = 0.33 *p* = 0.41
DB (mmHg)	81.5 ± 4.1	79.3 ± 3.1	81.7 ± 5.1	79.1 ± 6.0	78.7 ± 4.1	78.7 ± 3.1	*p* = 0.41 *p* = 0.42 *p* = 0.51 *p* = 0.49 *p* = 0.61
Energy intake (cal day)	1739.2 ± 129.2	1439.1 ± 119.2	1801.7 ± 113.1	1503.1 ± 112.2	1718.9 ± 112.1	1489.1 ± 123.1	*p* = 0.01 *p* = 0.02 *p* = 0.01 *p* = 0.48 *p* = 0.46
Carbohydrates (g/day)	181.9 ± 14.1	159.1 ± 19.1	192.7 ± 13.2	148.1 ± 13.2	179.7 ± 11.1	149.1 ± 10.1	*p* = 0.02 *p* = 0.03 *p* = 0.02 *p* = 0.41 *p* = 0.39
Fat (g/day)	61.5 ± 4.1	53.3 ± 3.2	79.7 ± 4.1	50.1 ± 7.2	76.9 ± 3.9	49.9 ± 4.1	*p* = 0.01 *p* = 0.01 *p* = 0.02 *p* = 0.33 *p* = 0.37
Protein (g/day)	84.5 ± 5.1	75.1 ± 9.2	86.7 ± 2.1	76.2 ± 7.2	88.0 ± 2.1	77.1 ± 8.1	*p* = 0.02 *p* = 0.02 *p* = 0.03 *p* = 0.60 *p* = 0.49
Monounsaturated fat (%)	34.5%	55.5%	34.9%	55.0%	35.1%	55.1%	*p* = 0.02 *p* = 0.03 *p* = 0.01 *p* = 0.37 *p* = 0.48
Polyunsaturated fat (%)	13.4%	22.7%	13.8%	22.0%	14.0%	23.0%	*p* = 0.02 *p* = 0.03 *p* = 0.04 *p* = 0.41 *p* = 0.42
Saturated fat (%)	52.1%	21.8%	53.5%	23.0%	50.9%	21.9%	*p* = 0.01 *p* = 0.02 *p* = 0.03 *p* = 0.38 *p* = 0.43
Physical activity (min/week)	121.1 ± 12.3	149.3 ± 21.1	123.1 ± 9.9	152.0 ± 23.2	122.1 ± 7.2	151.9 ± 18.1	*p* = 0.11 *p* = 0.22 *p* = 0.34 *p* = 0.30 *p* = 0.33

BMI: body mass index. SB: Systolic blood pressure. DB: Diastolic blood pressure WC: Waist circumference. (*) *p* < 0.05, in each genotype group. No differences between genotype groups.

**Table 2:** Biochemical parameters rs3774261 (mean ± S.D).

	AA (*n* = 117)		AG (*n* = 164)		AG (*n* = 80)		
	0 time	At 12 weeks	0 time	At 1 2 weeks	0 time	At 12 weeks	*p* Time AA Time AG Time GG Basal Genotype 12 weeks Genotype
Glucose (mg/dL)	102.1 ± 7.0	99.3 ± 7.1	101.5 ± 7.1	99.9 ± 5.1	101.1 ± 5.1	98.9 ± 4.1	*p* = 0.16 *p* = 0.19 *p* = 0.30 *p* = 0.45 *p* = 0.34
Total ch. (mg/dL)	204.1 ± 7.2	186.3 ± 4.2 *	208.7 ± 3.5	196.7 ± 6.0	205.1 ± 3.1	194.3 ± 7.2	*p* = 0.01 *p* = 0.22 *p* = 0.30 *p* = 0.41 *p* = 0.31
LDLch. (mg/dL)	127.3 ± 6.1	110.3 ± 7.2 *	129.1 ± 4.3	123.1 ± 8.2	127.1 ± 4.1	121.1 ± 9.1	*p* = 0.01 *p* = 0.32 *p* = 0.31 *p* = 0.35 *p* = 0.51
HDL-ch. (mg/dL)	52.8 ± 3.1	50.6 ± 4.0	51.9 ± 3.0	50.1 ± 2.1	52.3 ± 3.0	51.1 ± 3.1	*p* = 0.21 *p* = 0.32 *p* = 0.39 *p* = 0.25 *p* = 0.41
TG (mg/dL)	128.9 ± 11.1	93.1 ± 10.4 *	131.1 ± 6.2	121.1 ± 10.8	129.8 ± 4.2	123.9 ± 9.3	*p* = 0.03 *p* = 0.32 *p* = 0.30 *p* = 0.29 *p* = 0.31
Insulin (mUI/L)	13.1 ± 2.0	9.4 ± 1.9 *	13.3 ± 2.1	9.5 ± 1.1 *	12.7 ± 3.0	9.1 ± 3.0 *	*p* = 0.01 *p* = 0.02 *p* = 0.01 *p* = 0.45 *p* = 0.39
HOMA-IR	3.3 ± 0.6	2.0 ± 0.3 *	3.4 ± 0.5	2.1 ± 0.4 *	3.1 ± 0.2	1.8 ± 0.9 *	*p* = 0.01 *p* = 0.02 *p* = 0.02 *p* = 0.35 *p* = 0.41
CRP (mg/dL)	5.1 ± 1.0	3.8 ± 0.8 *	5.0 ± 2.9	4.9 ± 2.1	5.1 ± 3.1	5.0 ± 3.2	*p* = 0.01 *p* = 0.42 *p* = 0.41 *p* = 0.55 *p* = 0.19

Total Ch: Cholesterol. TG: Triglycerides LDL-ch: Low density lipoprotein cholesterol, HDL-ch: High density lipoprotein cholesterol. CRP: c reactive protein. HOMA-IR: Homeostasis model assessment. LDL: low density lipoprotein, HDL: High density lipoprotein. (*) *p* < 0.05, in each group. No statistical differences among genotypes in basal time or after 12 weeks. See significant deltas in the text.

**Table 3:** Serum levels of adipocytokines (mean ± S.D).

	AA (*n* = 117)		AG (*n* = 164)		AG (*n* = 80)		
	0 time	At 12 weeks	0 time	At 12 weeks	0 time	At 12 weeks	*p* Time AA Time AG Time GG Basal Genotype 12 weeks genotype
Adiponectin (ng/mL)	10.0 ± 3.1	21.6 ± 3.2 *	12.3 ± 3.3	14.4 ± 4.7	12.0 ± 2.8	15.5 ± 4.2	*p* = 0.01 *p* = 0.12 *p* = 0.31 *p* = 0.45 *p* = 0.11
Resistin (ng/mL)	5.1 ± 2.0	5.0 ± 1.6	5.2 ± 2.1	5.1 ± 1.1	5.3 ± 3.9	5.1 ± 3.2	*p* = 0.31 *p* = 0.32 *p* = 0.41 *p* = 0.45 *p* = 0.33
Leptin(ng/mL)	49.1 ± 9.1	12.9 ± 7.3 *	42.0 ± 7.1	22.1 ± 6.1 *	46.8 ± 4.2	19.4 ± 4.1 *	*p* = 0.01 *p* = 0.02 *p* = 0.03 *p* = 0.41 *p* = 0.31
Ratio Adiponectin/leptin	0.2 ± 0.1	1.7 ± 0.3 *	0.3 ± 0.2	0.6 ± 0.3	0.3 ± 0.2	0.7 ± 0.3	*p* = 0.02 *p* = 0.33 *p* = 0.34 *p* = 0.41 *p* = 0.51

(*) *p* < 0.05, in each group with basal values. No statistical differences among genotypes in basal time or after 12 weeks. See significant deltas in the text.
